# Estimating and Predicting Metal Concentration Using Online Turbidity Values and Water Quality Models in Two Rivers of the Taihu Basin, Eastern China

**DOI:** 10.1371/journal.pone.0152491

**Published:** 2016-03-30

**Authors:** Hong Yao, Wei Zhuang, Yu Qian, Bisheng Xia, Yang Yang, Xin Qian

**Affiliations:** 1 State Key Laboratory of Pollution Control and Resource Reuse, School of the Environment, Nanjing University, Nanjing, China; 2 School of Geography, Nantong University, Nantong, China; 3 Nanjing Institute of Environmental Sciences, Ministry of Environmental Protection of China, Nanjing, China; Purdue University, UNITED STATES

## Abstract

Turbidity (T) has been widely used to detect the occurrence of pollutants in surface water. Using data collected from January 2013 to June 2014 at eleven sites along two rivers feeding the Taihu Basin, China, the relationship between the concentration of five metals (aluminum (Al), titanium (Ti), nickel (Ni), vanadium (V), lead (Pb)) and turbidity was investigated. Metal concentration was determined using inductively coupled plasma mass spectrometry (ICP-MS). The linear regression of metal concentration and turbidity provided a good fit, with R^2^ = 0.86–0.93 for 72 data sets collected in the industrial river and R^2^ = 0.60–0.85 for 60 data sets collected in the cleaner river. All the regression presented good linear relationship, leading to the conclusion that the occurrence of the five metals are directly related to suspended solids, and these metal concentration could be approximated using these regression equations. Thus, the linear regression equations were applied to estimate the metal concentration using online turbidity data from January 1 to June 30 in 2014. In the prediction, the WASP 7.5.2 (Water Quality Analysis Simulation Program) model was introduced to interpret the transport and fates of total suspended solids; in addition, metal concentration downstream of the two rivers was predicted. All the relative errors between the estimated and measured metal concentration were within 30%, and those between the predicted and measured values were within 40%. The estimation and prediction process of metals’ concentration indicated that exploring the relationship between metals and turbidity values might be one effective technique for efficient estimation and prediction of metal concentration to facilitate better long-term monitoring with high temporal and spatial density.

## Introduction

Metals in surface water can seriously harm human and ecological health. The monitoring of these pollutants is extremely important [1–3]. However, the laboratory determination process of metals is lengthy and difficult [1], and on-site sampling is often resource limited. This results in sparse data sets with low temporal and spatial density.javascript:; Furthermore, It may be uncertain for the data obtained in the field due to analytical methods [4–6]. Water quality models are effective mathematical tools to interpret and predict the mixing, transport and transformation of pollutants. However, water quality models, which could be directly used to predict metal concentration, are scarce. For these reasons, it is important to refine techniques to enable efficient estimation and predictions of metal concentration to facilitate better long-term monitoring and accurate information following pollution incidents.

Because the high accuracy and rapid determination are provided by portable turbidity meters, turbidity is widely used to represent these water quality indicators by establishing mathematical models on pollutant concentration and turbidity values[7, 8]. The reliable quantitative relationship between total suspended solid (TSS) concentration and turbidity (T), including linear, exponential, power and polynomial functions, has been widely demonstrated both in laboratory and field studies[5, 9–20]. Metals are particle-bound pollutants in surface water with suspended solids associated with 60–97% of total metals in surface water[21–24]. Transported suspended solids absorb metals; therefore, turbidity could be used to detect the occurrence of these pollutants. The reliable relationship between metal concentration and suspended solids has been determined in some typical sources such as urban and road runoff[25–27]. However, until now, this relationship has never been tested in natural channels.

Turbidity is strongly influenced by the properties of transported sediment, such as shape, size, and mineral composition[7, 14, 28]. Metals in surface water originate from natural processes such as atmospheric deposition and geological weathering, as well as from anthropogenic activities. Sediments from a range of sources may display different properties, and the contributions of these sources can vary between different watersheds. In addition, discharges of cross sections and the particle size distribution of suspended solids can also affect the properties of suspended solids in surface water. This has been well documented in previous studies [28–32] which means that the relationship between turbidity, TSS, and metals might vary spatially, making information about these variations important for decision makers involved in water resource management[33–37].

Thus, the aims of the study were as follows: (1) to explore the relationship between turbidity and metals based on an observation in two rivers with different ecological functions and water quality conditions; (2) to apply the relationship between turbidity and metals to estimate metal concentration with high temporal density using online turbidity data; (3) to attempt to predict metal concentration with high temporal and spatial density using the relationship between turbidity and metals by means of water quality modeling to improve understanding of turbidity-based estimation and prediction models for these potential particle-bound pollutants.

## Materials and Methods

### Ethics statement

No specific permits were required for the described field studies which did not involve endangering or protected species.

### Study area

**Fig 1:**
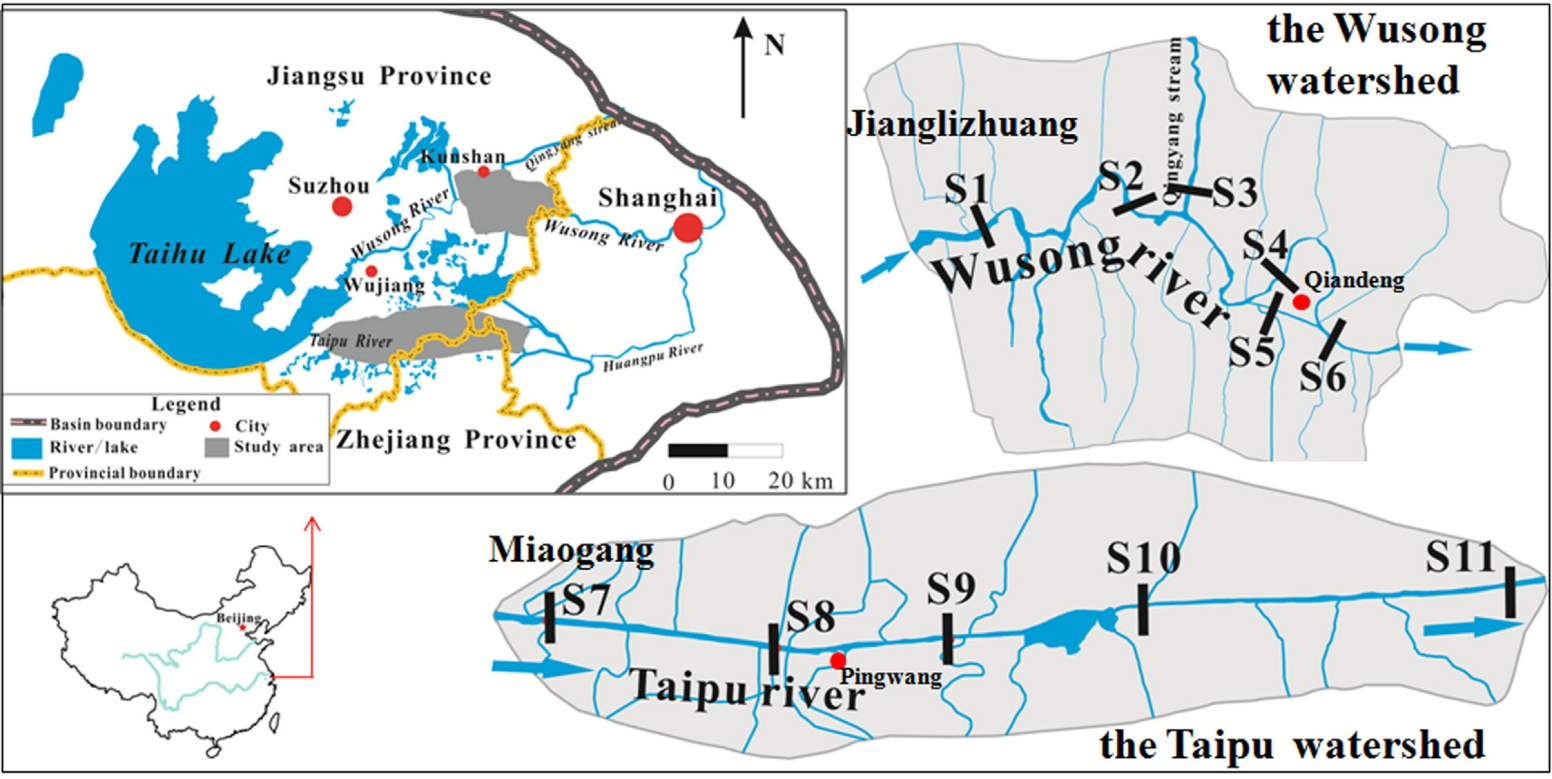
Location map for the study area and the 11 observation sites.

The Wusong River and the Taipu River, situated to the east of the Taihu Basin (Fig 1), were the two rivers studied in this paper. The eastern part of the Taihu Basin is one of the most developed regions in China, and large amounts of wastewater containing heavy metals is discharged into surface waters in the basin every year[38–40].

The catchment areas of the Wusong River and the Taipu River are 323 km^2^ and 256 km^2^ respectively. The Wusong watershed is located in Kunshan, Jiangsu province. The Wusong River is the trunk of the watershed. The segment in the studied area is 35 km long and approximately 80 m wide. The Qingyang stream is the main tributary of the Wusong River. This watershed is densely populated and has many economic activities. The Wusong River mainly serves as an industrial area, with more than 300 industrial sites along its banks [38]. The automatic hydrological determination station along the Wusong River only exists at Jianglizhuang station at S_1_ (Fig 1).

The Taipu River is the main river in the Taipu watershed. It is 70 km long and averages 200 m wide. The river is relatively clean and has multiple functions including flood prevention, irrigation, and shipping. It also serves as the source of drinking water for Shanghai. Miaogang station at S_7_ is the only automatic hydrological determination station along the Taipu River (Fig 1).

### Framework of the methodology

**Fig 2:**
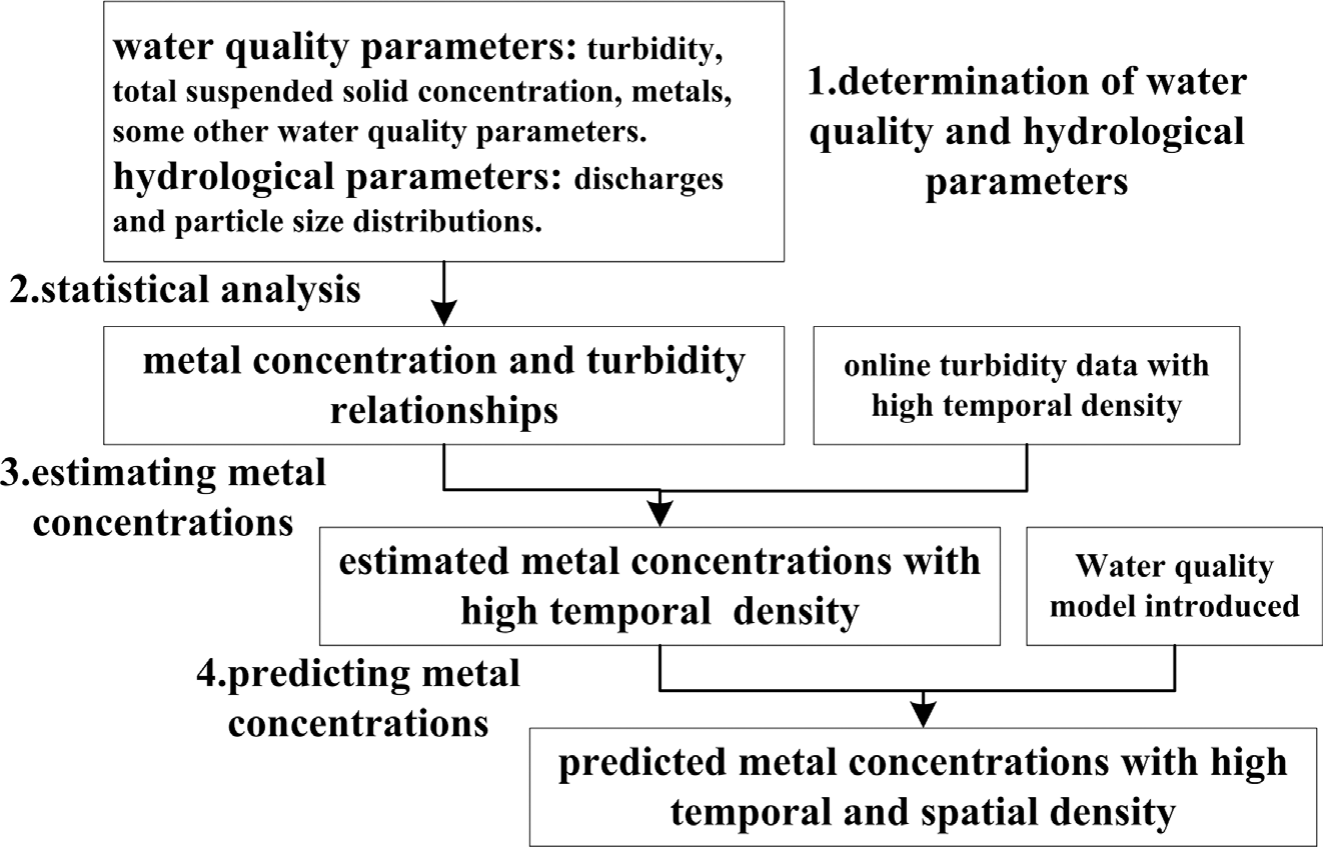
Framework of estimating and predicting metal concentration.

The proposed method to estimate and predict metal concentration using online turbidity values and water quality models includes four different steps (Fig 2): (1) determining water quality and hydrological parameters, including turbidity, total suspended solid concentration, metals, some other water quality parameters, discharges and particle size distributions, (2) statistical analysis of the metal concentration and turbidity relationship, (3) estimating metal concentration using online turbidity data with high temporal density, (4) predicting metal concentration by water quality modeling and obtaining metal concentration with high temporal and spatial density. Detailed explanations of these four components are provided below.

### Determination of water quality and hydrological parameters

#### Sampling

Eleven sampling sites were selected at regular intervals along the two rivers (see Fig 1). Six of these (S_1_-S_6_) were in the Wusong River, and five (S_7_-S_11_) were in the Taipu River. Samples were collected once a month from January 2013 to June 2014 and were all performed in dry weather to eliminate extreme conditions such as floods or snowfall. All samples were taken with 5 L Plexiglas samplers on bridges, with a sampling depth of 20 cm below the water surface.

#### Turbidity (T)

Turbidities were all monitored in situ using an HACH 2100Q Portable Turbidimeter and reported in NTU. The turbidimeter was calibrated using formazine prior to each sample collection. Three sub-samples were taken from the mixed sample from across the river, and three turbidity values were read for each sub-sample, providing a total of nine turbidity measurements for each sample site. The average value of these nine turbidity measurements was used in our analysis.

#### Total suspended solid (TSS) concentration

Total suspended solid (TSS) concentration was measured in the laboratory by filtering 500 mL water samples through a membrane with a 0.45 μm pore dried to a constant weight at 105°C according to the national standard method of China [1, 41]. The filter and retained sediment were also dried to a constant weight at the same temperature, and the TSS value was calculated according to the added weight of the membrane. An average value of three parallel subsamples was then used to provide the TSS concentration for each sample.

#### Metals

Metal concentration was determined using inductively coupled plasma mass spectrometry (ICP-MS), and two forms of metals, including dissolved metals and total metals, were measured.

Each 500 mL sample was filtered through a 0.45 μm pore membrane. To avoid contamination from the filtering apparatus, the first 200 mL of filtered water was discarded in each case. The filtered sub-samples were used to analyze dissolved metal concentration directly using ICP-MS.

The unfiltered water was used to analyze total metal concentration. Four 25 mL sub-samples of the unfiltered water in the 1 L polytetrafluoroethylene bottle were taken, mixed and then digested referring to the acid digestion of aqueous samples and extracts for total metals under analysis by ICP spectroscopy (EPA method 3010) [42]. The concentration of five metals (aluminum (Al), titanium (Ti), nickel (Ni), vanadium (V), lead (Pb)), which prefer to be in particle-phase in water, was analyzed using ICP-MS.

From these measurements, particulate metal concentration (>0.45 μm) can be approximated using total metal concentration minus dissolved metal concentration.

#### Other water quality parameters

Other water quality parameters, including PH, electrical conductivity, dissolved oxygen, biochemical oxygen demand, chemistry oxygen demand, ammonia, nitrate, organic nitrogen, orthophosphate, dissolved phosphorus and total phosphorus, were analyzed using the standard methods recommended by China’s Ministry of Environmental Protection [43]. Again, the average value taken from three sub-samples was used to provide water quality parameter concentration for each sample site. These water quality parameters were used for parameter calibration and results verification in water quality model evaluations.

#### Discharges and particle size distributions

Hydrological parameters of cross sections were determined using River Surveyor, the acoustic Doppler profiler by SonTek. In the Wusong River, the hydrological parameters were measured at the cross sections of S_1_ and S_6_ (S_9_ and S_11_ in the Taipu River).

The particle size distributions of all collected samples were analyzed using a Mastersizer 2000 particle size analyzer.

### Statistical analysis of the metal concentration and turbidity relationship

Several statistical regression models were tested to link the metal concentration with the turbidity values. The linear regression model, which has been widely used in analyzing the turbidity to pollutant concentration relationship in previous studies[9, 15, 20, 44], provided the best results with the lowest uncertainty (highest R^2^). Linear regression of TSS-T, Al-T, Ti-T, Ni-T, V-T, Pb-T was described separately in the two rivers.

### Estimating metal concentration using online turbidity data

The online turbidity data were collected from the automatic hydrological determination station. The automatic turbidity was measured using an analyzer that measures five water quality parameters; in China, automatic hydrological determination stations are generally equipped with an analyzer to measure five water quality parameters, and the equipment can rapidly determine water temperature, pH, dissolved oxygen, conductivity, and turbidity.

The relationship of metal concentration and turbidity was applied to estimate metal concentration using online turbidity data. The relative errors between the measured metal concentration and the estimated values were used to judge whether the relationship of metals and turbidity were credible and reliable. The relative errors were calculated using the following formula:

$$\varepsilon =\frac{\left|C_{\text{m}}-C_{\text{e}}\right|}{C_{\text{m}}}\times 100\%$$

where *Ɛ* denotes the relative error between the measured value and the estimated value, *C*_m_ denotes the measured concentration and *C*_e_ denotes the estimated concentration using the relationship of metal concentration and turbidity.

### Predicting metal concentration by water quality modeling

Based on the hydrological characteristics of the study area, we selected the WASP 7.5.2 (Water Quality Analysis Simulation Program) model, which was developed by USEPA and is one of the most popular water quality models [40, 45, 46].The EUTRO module in WASP can interpret the transport and fate of traditional pollutants, including total suspended solids.

The metal concentration prediction procedure includes four parts: (1) applying the TSS-T relationship and estimating TSS concentration using online turbidity data; (2) using the water quality model introduced to predict TSS concentration downstream; (3) applying the TSS-T relationship to calculate turbidity data downstream using predicted TSS concentration and obtaining predicted turbidity; (4) finally, using the relationship of metal concentration and turbidity to predict metal concentration downstream with short time intervals.

Similarly, the relative errors between the measured values of metal concentration and the predicted values were calculated using the following formula:

$$\varepsilon =\frac{\left|C_{\text{m}}-C_{\text{p}}\right|}{C_{\text{m}}}\times 100\%$$

where *Ɛ* denotes the relative error between the measured value and the predicted value, *C*_m_ denotes the measured concentration and *C*_p_ denotes the predicted metal concentration.

## Results and Discussion

### Metal concentration and turbidity relationships

#### Plotted diagram of the different element concentration and turbidity

The samples collected on the two rivers from January 2013 to December 2013 were used to explore the relationship of metal concentration and turbidity. The concentration of total metals and TSS was introduced. For each element, 72 data sets of the Wusong River and 60 data sets of the Taipu River were analyzed. Generally, the different element concentration and turbidity in the Wusong River were higher than those in the Taipu River. The descriptive statistics of the variables from all the water samples of the two rivers are listed in Table 1.

**Table 1:** Descriptive statistics of the variables in all water samples from the two rivers.

Study area	in the Wusong River (n = 72)				in the Taipu River (n = 60)			
	Mean	Min*	Max*	SD*	Mean	Min*	Max*	SD*
**T(NTU)**	44.143	8.790	99.190	20.958	23.016	5.205	44.513	9.473
**TSS(mg/L)**	45.221	11.400	79.667	15.937	26.417	2.400	52.892	11.598
**Al(mg/L)**	2.758	0.505	6.114	1.389	1.638	0.556	3.570	0.639
**Ti(mg/L)**	0.052	0.007	0.120	0.026	0.023	0.010	0.038	0.007
**Ni(mg/L)**	0.030	0.011	0.064	0.012	0.012	0.006	0.017	0.003
**V(mg/L)**	0.012	0.006	0.022	0.004	0.007	0.005	0.010	0.001
**Pb(mg/L)**	0.105	0.012	0.241	0.050	0.036	0.007	0.075	0.017

* Abbreviations: SD denotes the standard deviation; Min denotes the minimum value of the data set; Max denotes the maximum value of the data set.

**Fig 3:**
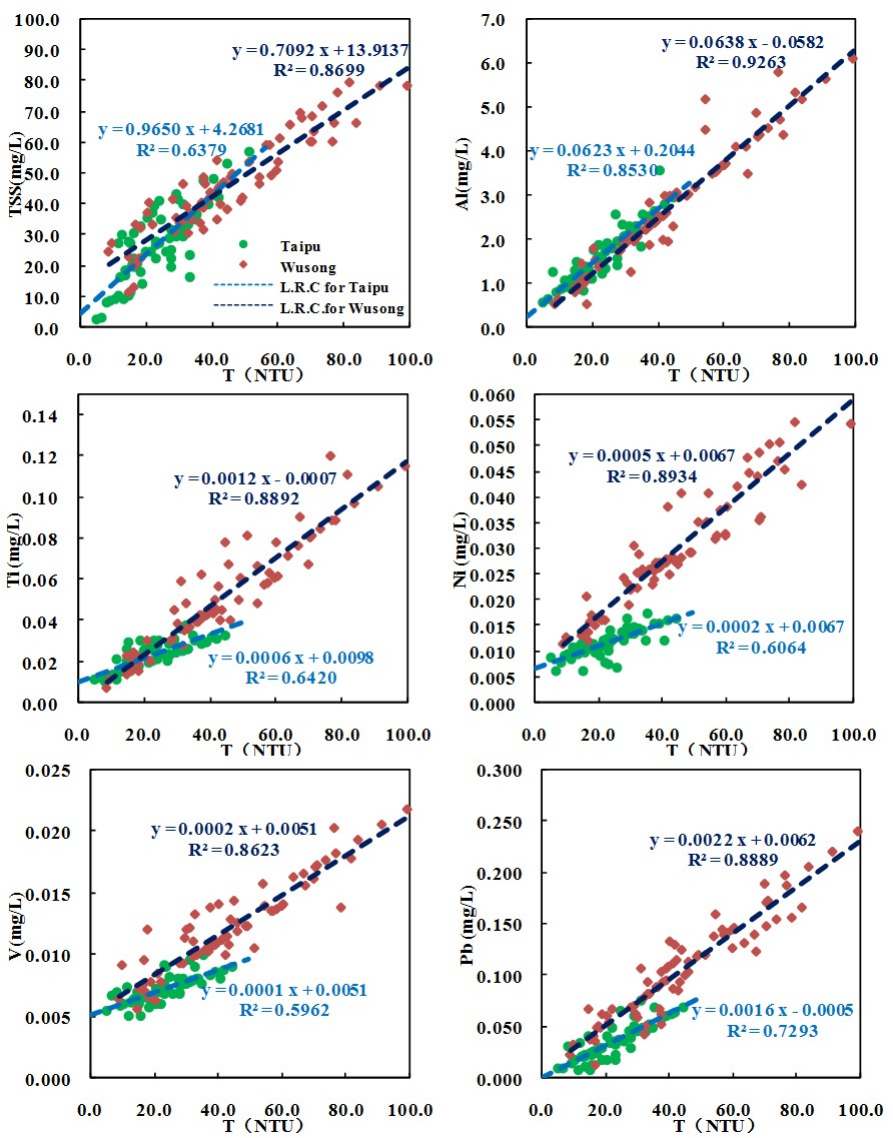
Plotted diagram of concentration of TSS and metals with turbidity values.

The results are presented in Fig 3. The key for each symbol used in Fig 3 is provided in the TSS-T plotted diagram. Green dots denote the different element concentration of samples from the Taipu River, and red boxes denote those from the Wusong River. The slopes and R^2^ values of the linear regression curves for the relationship between the different element concentration and turbidity are also provided in Fig 3; L.R.C. denotes linear regression curve for the two variables in the diagram. Light blue curves represent that of the Taipu River, and deep blue curves represent the Wusong River. In all equations of the linear regression curves, y denotes the concentration of the relevant metal (all units in mg/L), and x denotes the turbidity values (all units in NTU). R^2^ denotes the fitting coefficient in the corresponding linear model.

#### Total suspended solids and turbidity

Good linear relationship of TSS-T was found in the two rivers (Fig 3). The 72 data sets collected along the Wusong River yielded R^2^ = 0.8699, and the 60 data sets collected along the Taipu River yielded R ^2^ = 0.6379. As shown in Table 2, the two kinds of linear relationship are comparable to those reported in previous investigations. Table 2 also provides the slopes and R^2^ values from previous studies to aid the discussion.

**Table 2:** Reported TSS-T regression models.

Study area	Range of T	Equation of TSS-T^#^	R^2^	Reference
Five catchments, Germany	0–114 NTU	*C* = 1.86*T	0.89	[20]
Lowland streams, Puget	0–240 NTU	*C* = 0.15*T^1.32^	0.96	[12]
Laboratory	0–60000 NTU	*C* = 0.00065*T+2.78	0.79	[15]
Elbe River, Germany	0–500 NTU	*C* = 0.0103*Q^0.7384^*T+15.2	0.97	[14]
Lartrobe River, Australia	>800 FAU	*C* = 2.70*T-990	0.88	[9]
Lartrobe River, Australia	<800 FAU	*C* = 0.73*T+32.15	0.93	[9]
Tidal Saltmarsh, Northeast US	0–50 FTU	*C* = 1.584*T+2.107	0.83	[11]
Sauerbier creek, Australia	0–1000 NTU	*C* = 17.36*(T/100)^2^–81.615*(T/100)+178.96	0.78	[13]
Clear Creek, US	0–70 NTU	Log_10_ *C* = 1.25*Log_10_T	0.87	[18]
One mountainous catchment, French	0–60 g/L SiO_2_	*C* = 0.032*T^2^+0.262*T	-	[5]
Yellow River in Atlanta, US	0–300 FNU	*-*	0.90	[28]
Wusong River, China	9–99 NTU	*C* = 0.709*T+13.914	0.87	this study
Taipu River, China	5–45 NTU	*C* = 0.965*T+4.268	0.64	this study

^#^In all equations, *C* denotes the concentrations of the suspended solids, and the unit was mg/L.

The slopes of the two TSS-T linear regression equations, which were 0.7092 in the Wusong River and 0.9650 in the Taipu River, were similar. The similar relationship displayed by TSS-T is thought to be due to the similarities in natural processes, i.e., similar geographical and climatic conditions in the two watersheds, which are only approximately 20 km from each other [20]. The two slopes were also close to those of the TSS-T linear regression models in the Lartrobe River of Australia (T<800 FAU) [9]; a tidal salt marsh in the northeast US [11], and five neighboring catchments in Germany [20], where all values were in the vicinity of 1.

#### Metals and turbidity

The concentrations of the five metals fit well with the turbidity values, as shown in Fig 3. The linear regressions of metal concentrations and turbidity provided a good fit with R^2^ = 0.86–0.93 for 72 data sets collected along the Wusong River and R ^2^ = 0.60–0.85 for 60 data sets collected in the Taipu River. The good linear relationship implies that these metal concentrations could be approximated using these regression equations.

**Fig 4:**
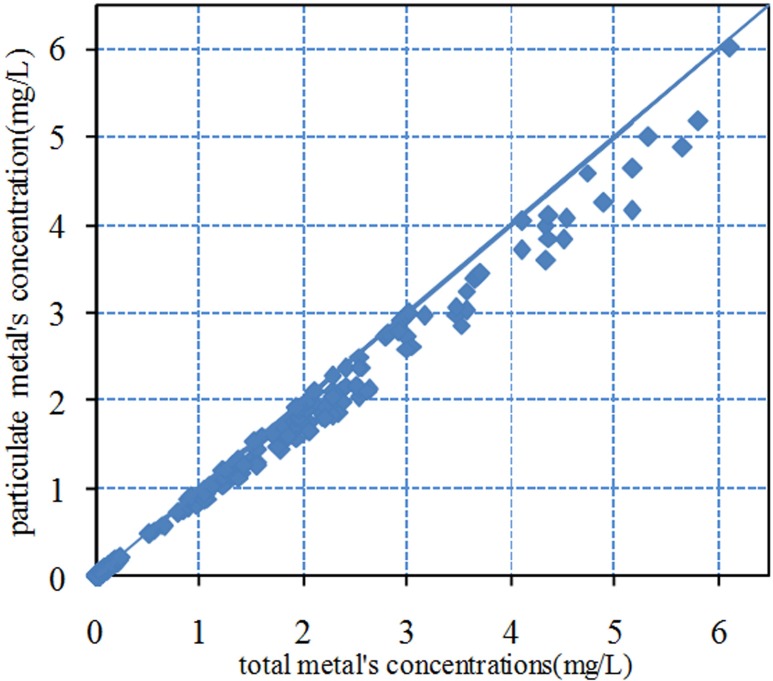
The plotted graph of concentration of particulate metals and total metals.

All presented good linear relationship, leading to the conclusion that the occurrence of the five metals are directly related to suspended solids, which could be supported by the relationship of particulate metal (PM) concentrations and total metal (TM) concentrations. Metals in surface water exist in the form of particulate metals and dissolved metals. Fig 4 provides the concentrations of PM and TM from all observations (660 data sets) made in 2013, and both concentrations were similar in both rivers. As the figure demonstrates, metals in the two rivers are particle-bound pollutants with suspended solids associated with 81–98% of total metals in surface water, and these proportion values were also close to those in previous studies[21–24].

Of the five metals, the slopes of the Al-T linear regression equations, which were 0.0638 in the Wusong River and 0.0623 in the Taipu River, were very similar to each other. This consistency verified that Al in the surface water of the two rivers existed in similar forms, and this result was consistent with what was found by Berndtsson [27]. The similar relationship displayed by Al-T is thought to be due to the properties of Al in surface water. The element closely adhering to suspended solids in the colloidal state and in surface water displayed similar transport properties with suspended solids [27].

Ti-T, Ni-T, V-T and Pb-T had similar spatial distributions. In the Wusong River, the slopes and R^2^ of the linear regression equations were higher than those in the Taipu River. This spatial difference may be due to the source characteristics of the four metals, which primarily originate from industrial emissions [39, 47]. Thus, the uncertainty of sources might add to the spatial uncertainty of the Ti-T, Ni-T, V-T and Pb-T relationship in the Taipu River, whereas in the industrial river, the four kinds of relationship were consistent.

These spatial variations of the Ti-T, Ni-T, V-T and Pb-T relationship might originate from differences in sources of solids and hydrological conditions. Human activities are variable in the two rivers; thus, suspended matter in surface waters may come from different sources with spatial fluctuation characteristics in diverse watersheds [33, 44]. In the industrial watershed of the Wusong River, suspended solids are thought to primarily originate from industrial emissions in all seasons [39]. The Taipu watershed is influenced by several different sources, which may be more prone to seasonal and spatial variations [39]. It is thought that the seasonal and spatial variations in the dominant source of suspended solids cause this lack of certainty in the cleaner watershed of the Taipu River.

Hydrological parameters also varied in the two rivers and led to a diverse range of transported sediment properties, such as particles’ shape and size. These are presented for each river in Table 3. Turbidity has been found to be strongly influenced by particle properties[28, 48]. The discharges and flow rates in the Taipu River are much higher than those in the Wusong River and, as shown in Table 3, the particle sizes are larger. Furthermore, variations in particle sizes between the different sampling sites in the Taipu River (displayed by the standard deviations) were more significant than those in the Wusong River. These variations can be explained in part by the diversity of suspended solid sources in the Taipu River.

**Table 3:** Hydrological conditions and particle size distribution in the two rivers.

Study area	in the Wusong River				in the Taipu River			
	Mean	Min*	Max*	SD*	Mean	Min*	Max*	SD*
discharge**	48.448	32.350	68.440	15.821	352.520	230.310	483.850	112.013
flow rate**	0.188	0.170	0.210	0.021	0.400	0.300	0.550	0.115
d***	10.646	6.082	14.045	2.755	13.768	2.457	21.559	4.566

* Abbreviations: SD denotes standard deviation; min denotes the minimum value; max denotes the maximum value.

** The discharge and flow rate values in the Wusong River denote the variables measured at S_1_ and S_6_ (n = 24), those in the Taipu River denote the variables observed at S_9_ and S_11_ (n = 24). The unit of discharge was m^3^/s, and the unit of flow rate was m/s.

***d denotes the particle diameter of surface weighted mean. The unit was um.

Although spatial variations existed in the two rivers over the Al-T, Ti-T, Ni-T, V-T and Pb-T relationship, the good linear regression demonstrated that the trend of turbidity in surface water is consistent with that of the five metals; therefore, Al, Ti, Ni, V and Pb concentrations could all be approximated by T values in the two rivers.

### Metal concentration estimation

The online turbidity data of S_1_ and S_7_ sites from January 1 to June 30 of 2014 were separately collected from the Jianglizhuang automatic hydrological determination station and the Miaogang hydrological determination station. The automatic turbidity in the two sites was measured by the five water quality parameters analyzer. No significant differences could be observed between the online data and field measurements using the HACH 2100Q Portable Turbidimeter. The interval of online turbidity data was 2 hours. The online turbidity values at S_1_ were 15.5–29.0 NTU, the average was 21.4 NTU and the standard deviation was 3.4. The online turbidity values at S_7_ were 8.1–24.9 NTU, the average was 13.6 NTU and the standard deviation was 3.2.

The linear regression equations of metal concentrations and turbidity were applied to estimate metal concentrations using online turbidity data from January 1 to June 30 of 2014. Metal concentrations with high temporal density at S_1_ and S_7_ were estimated by online turbidity.

**Fig 5:**
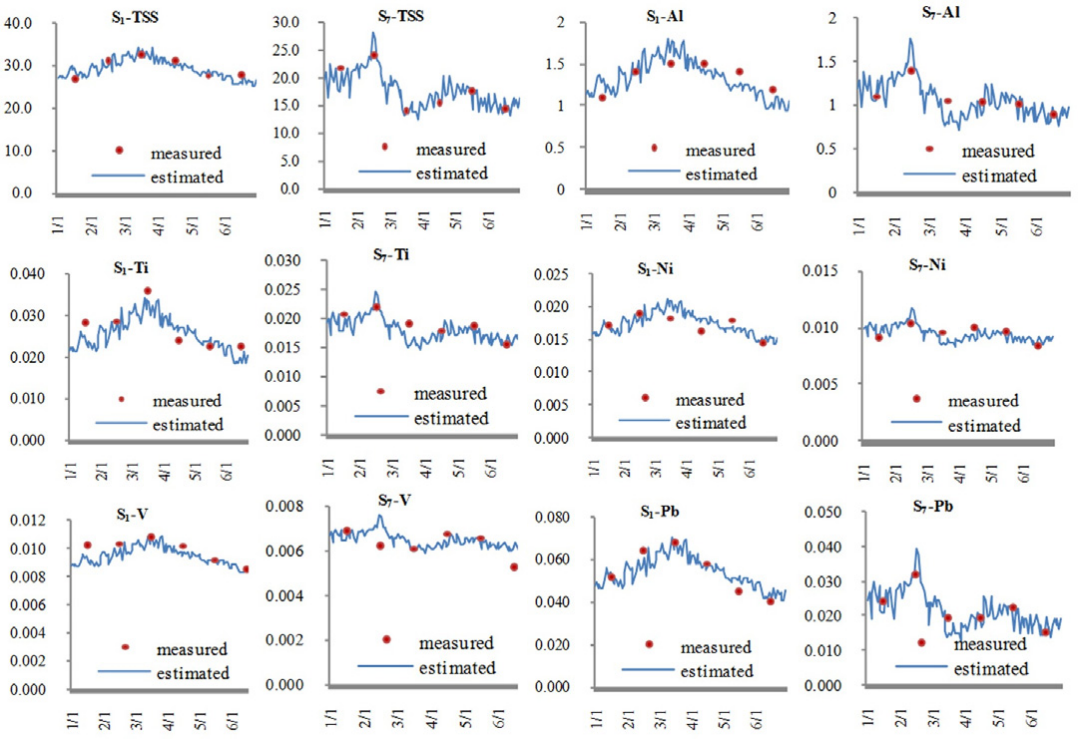
Measured and L.R.C.-estimated concentrations of metals and TSS at S1 and S7.

Fig 5 describes the estimated concentrations and the measured values of TSS and the five metals at S_1_ and S_7_. The horizontal coordinate denotes the data investigated, and the vertical coordinate denotes the measured and estimated concentrations of the elements analyzed. The units of all the vertical coordinates are in mg/L. The relative errors between the measured values of elements’ concentrations and the estimated values were calculated. All the relative errors between the estimated and measured values were within 30% (Fig 5). In the industrial river, the max relative errors between the element estimated concentrations and measured values were 9% of TSS, 20% of Al, 18% of Ti, 14% of Ni, 8% of V and 16% of Pb; in the cleaner river, the max relative errors were 20% of TSS, 25% of Al, 19% of Ti, 12% of Ni, 22% of V and 28% of Pb. This indicated that the estimated metal concentrations using linear regression curves were reliable in the Wusong River and the Taipu River, and the concentrations of Al, Ti, Ni, V and Pb could be approximated by online turbidity data in the two rivers.

### Metal concentration predictions

#### Water quality model evaluation and TSS prediction

The EUTRO and one-dimensional river network modules in WASP were used to reflect hydrodynamic characteristics and water quality response to natural and anthropogenic contamination in the two rivers [40]. Hydrological parameters input in the models were collected using River Surveyor. Estimated TSS concentrations by online turbidity data using TSS-T relationship at S_1_ and S_7_ were also input in the two models.

**Fig 6:**
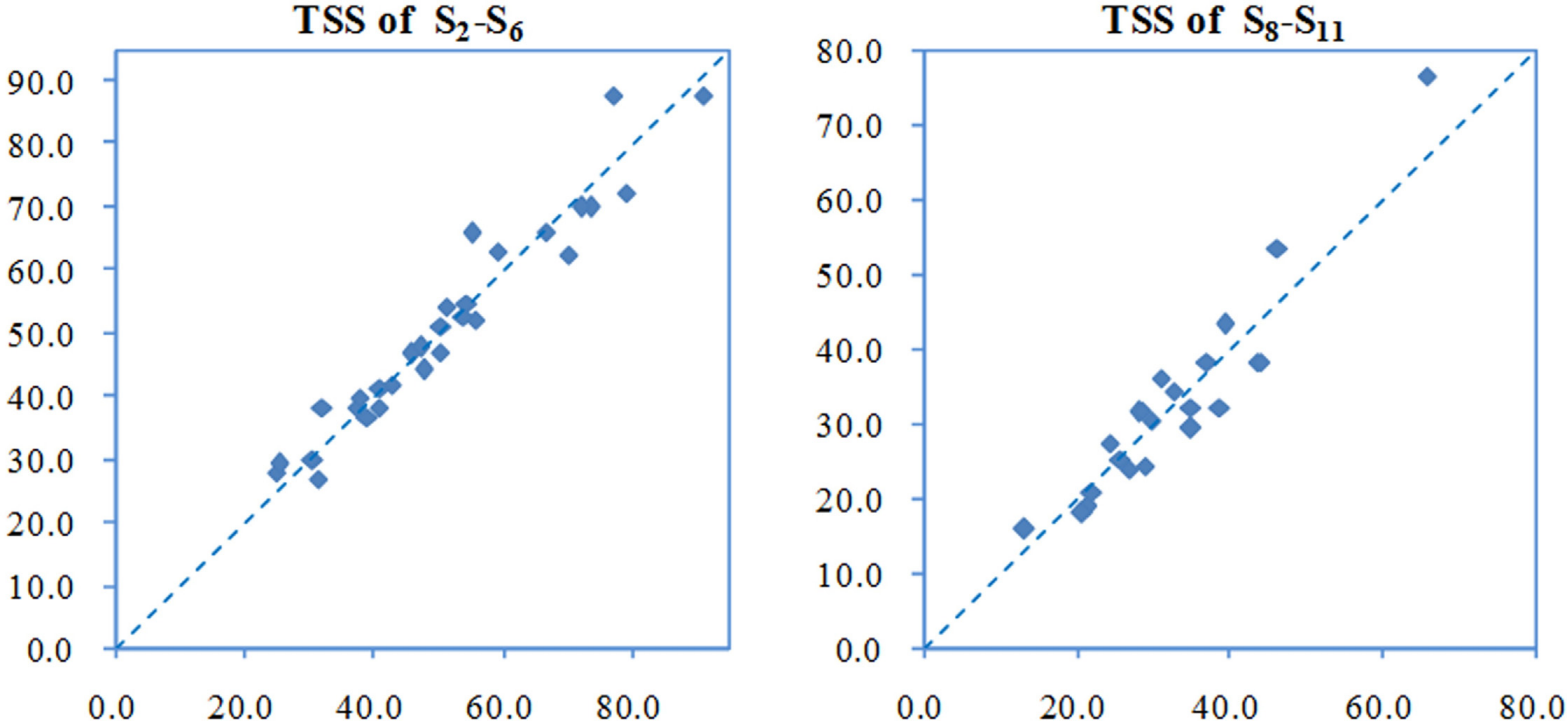
Measured and WASP-predicted concentrations of TSS at S2-S6 and S8-S11.

Model parameters were estimated from the recommended values provided in the WASP user manual. Through simulation tests, unreasonable assumptions were iteratively modified until the relative errors between the predicted and measured values (including dissolved oxygen, biochemical oxygen demand, chemistry oxygen demand, ammonia, nitrate, organic nitrogen, orthophosphate, dissolved phosphorus, total phosphorus and TSS) were within 15% (Fig 6), which indicated that the deviation between observed and predicted values was small and that the model results were acceptable for the two rivers[40]. This evaluation process ensured that the water quality models was stable and appropriate for predicting TSS concentration for the potential scenarios in the two rivers.

Fig 6 provides the predicted concentrations and the measured values of TSS at S_2_-S_6_ of the Wusong River and S_8_-S_11_ of the Taipu River from January to June, 2014. In the figure, the horizontal coordinate denotes measured TSS concentrations, the vertical coordinate denotes predicted TSS, and the units are mg/L. Of the 30 data sets in the industrial river, the max relative error between predicted TSS concentration and measured values was 14%; of the 24 data sets in the Taipu River, the max relative error was 18%.

#### Metal concentration predictions

Following the method outlined in the second section, TSS-T relationship was applied to calculate turbidity data downstream using predicted TSS concentration, and then the linear regression curves of the five metal concentrations and turbidity were used to predict metal concentrations downstream with short time intervals from January 1 to June 30 of 2014.

**Fig 7:**
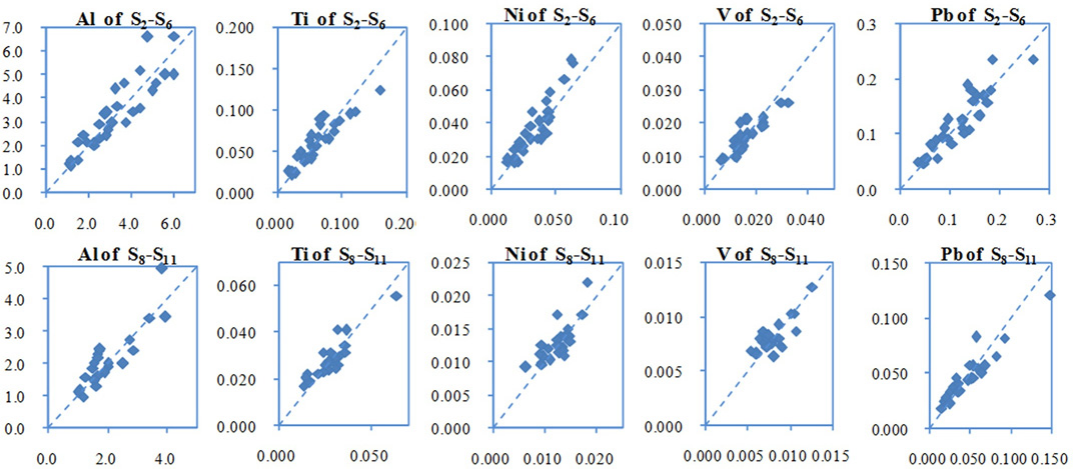
Measured and WASP-predicted concentrations of metals at S2-S6 and S8-S11.

Similarly, Fig 7 describes the predicted concentrations and the measured values of the five metals at S_2_-S_6_ and S_8_-S_11_. In the figure, the horizontal coordinate denotes measured values and the vertical coordinate predicted values. The units of all the coordinates are mg/L.

The relative errors between the measured concentrations and the predicted values were calculated. The max relative errors were 32% of Al, 21% of Ti, 16% of Ni, 11% of V and 34% of Pb in the Wusong River, and in the Taipu River, the max relative errors were 37% of Al, 15% of Ti, 22% of Ni, 17% of V and 38% of Pb. All the relative errors between the predicted and measured values were within 40% (Fig 7). The concentrations of Al, Ti, Ni, V and Pb could be approximately predicted by water quality model using online turbidity data and the linear relationship of metals and turbidity values.

**Fig 8:**
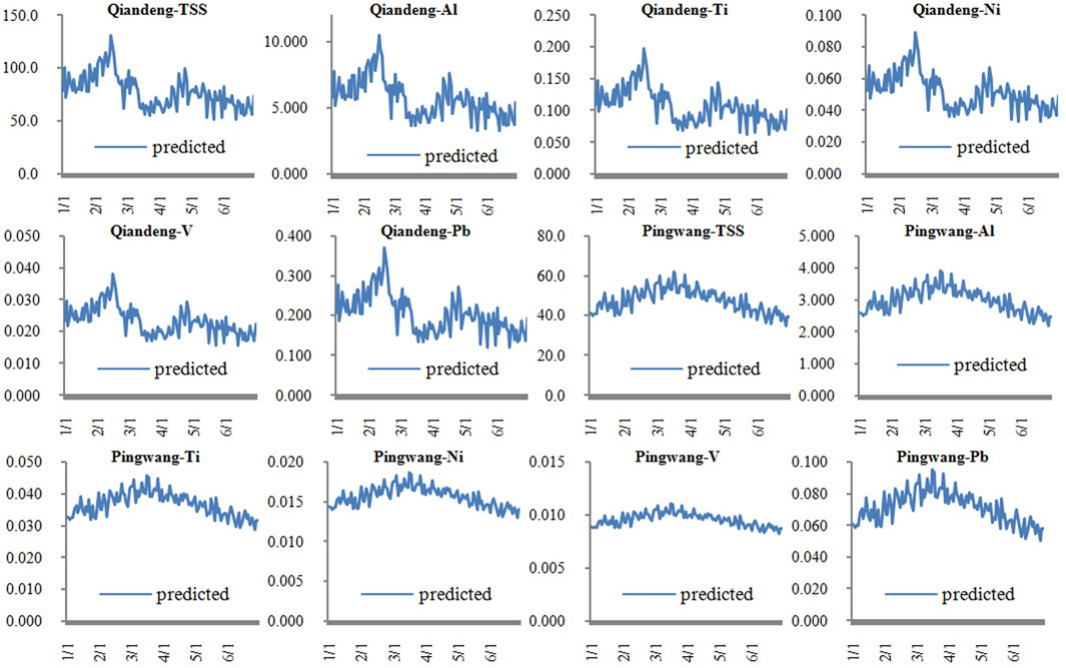
Predicted concentrations of metals at the Qiandeng town and the Pingwang town.

Thus, metal concentrations at any site downstream of the two rivers could be predicted by online turbidity of S1 and S7 and water quality modeling. As an example, Fig 8 describes the predicted metal concentrations from January 1 to June 30 of 2014 at the Qiandeng town of the Wusong River and the Pingwang town of the Taipu River, which are approximately located at S5 and S8 and are the most polluted regions of the two watersheds respectively (Fig 1). In the figure, the units of all the vertical coordinates are in mg/L. The prediction process of Al, Ti, Ni, V and Pb with high temporal and spatial density was practicable and credible in the two rivers (Fig 8).

## Conclusions

Turbidity (T) has been widely used to detect the occurrence of pollutants in surface water. Using data collected from January 2013 to June 2014 at eleven sites along two rivers feeding the Taihu Basin, China, relationship between the concentrations of five metals (Al, Ti, Ni, V and Pb) and turbidity were investigated.

The linear regressions of metal concentrationsand turbidity provided a good fit with R^2^ = 0.86–0.93 for 72 data sets collected in the industrial river and R ^2^ = 0.60–0.85 for 60 data sets collected in the cleaner river. All the regressions presented good linear relationship, leading to the conclusion that the occurrence of the five metals are directly related to suspended solids, and these metal concentration could be approximated using these regression equations.

Thus, the linear regression equations of metal concentrations and turbidity were applied to estimate metal concentrations using online turbidity data and predict metal concentrations downstream by means of water quality models of the two rivers from January 1 to June 30 of 2014. In the prediction, the WASP 7.5.2 (Water Quality Analysis Simulation Program) model was introduced to interpret the transport and fate of total suspended solids. All the relative errors between the estimated and measured values were within 30%, and all the relative errors between the predicted and measured values were within 40%.

The estimation and prediction process of Al, Ti, Ni, V and Pb with high temporal and spatial density was practicable and credible in the two rivers. Exploring the relationship of metals and turbidity values might be one effective technique for efficient estimation and prediction of metal concentrations to facilitate better long-term monitoring and accurate information following pollution incidents.
